# The first report of M918L ( *super‐kdr* ) and L932F voltage‐gated sodium channel mutations in the English grain aphid ( *Sitobion avenae* )

**DOI:** 10.1002/ps.71178

**Published:** 2026-08-05

**Authors:** Virgile Ballandras, Louise McNamara, James C Carolan, Tom Ruttink, Stephen Byrne

**Affiliations:** ^1^ Teagasc Crops Research Centre Oak Park Ireland; ^2^ Department of Biology Maynooth University Maynooth Ireland; ^3^ Flanders Research Institute for Agriculture, Fisheries and Food (ILVO), Plant Sciences Unit Melle Belgium; ^4^ Department of Plant Biotechnology and Bioinformatics, Faculty of Sciences Ghent University Ghent Belgium

**Keywords:** English grain aphid, knockdown resistance, pyrethroids, *super‐kdr*

## Abstract

**BACKGROUND:**

The grain aphid, *Sitobion avenae* Fabricius (Hemiptera: Aphididae), is a vector of yellow dwarf viruses and an important pest of cereals. In a European context, it is the only cereal aphid known to have developed knockdown resistance (*kdr*). In this study, we genetically screened key regions of the voltage‐gated sodium channel (VGSC) known to harbour resistance‐conferring mutations. Specimens were sampled across multiple locations and years in Ireland, enabling detection of individuals carrying the L1014F mutation and screening for additional mutations, including those associated with *super*‐*kdr*.

**RESULTS:**

The frequency of the L1014F substitution in samples from Irish suction towers is consistent with frequencies reported in other studies from Ireland and the UK, and we found no strong evidence of an increase in *kdr* frequency despite the greater reliance on pyrethroids. Importantly, the analysis identified two additional VGSC substitutions corresponding to M918L and L932F, which have been implicated in reduced pyrethroid sensitivity in other insect species. This is the first report globally of these mutations in *S*. *avenae*.

**CONCLUSION:**

We identified a *super‐kdr* variant (M918L) and an L932F variant in the grain aphid *S*. *avenae*. Because these were detected in archived samples, their impact on in‐field resistance in this species is currently unknown; however, based on observations in other aphid species, including cereal aphids, they likely influence resistance phenotypes. Regular monitoring of the incidence and spread of known and emerging insecticide‐resistant clonal lineages is essential to preserve the effectiveness of our limited insecticide arsenal and to determine when field efficacy may be insufficient for pest control. © 2026 The Author(s). *Pest Management Science* published by John Wiley & Sons Ltd on behalf of Society of Chemical Industry.

## Graphical abstract

The study has identified mutations in the voltage gated sodium channel of the English grain aphid (*Sitobion avenae*) at M918 and L932, which have not been previously reported in this species.

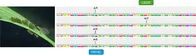

## 1 INTRODUCTION

Aphids are economically significant agricultural pests that cause damage to a wide range of crop species. Aphids damage crops directly by feeding and indirectly through vectoring plant viruses including yellow dwarf viruses (YDV) in cereals. Aphid control has relied heavily on the use of insecticides, particularly pyrethroids. Pyrethroids are a class 3A insecticide that act on voltage‐gated sodium channels (VGSC), disrupting insect nerve function, and causing paralysis and death in affected insects (reviewed in Bass and Nauen^1^). The number of available insecticides for arable crops has decreased in recent years, exacerbating selection pressure for resistance. This is particularly concerning for cereal crops because, in some cases, there may be no alternative mode of action available to rotate against pyrethroids.

Several mechanisms underlie pyrethroid resistance in insects, including reduced cuticular penetration,^2^ metabolic resistance^3^ and target‐site resistance.^4^ Target‐site resistance mechanisms have been linked to significant field control failures; the most common target‐site resistance mechanism is known as knockdown resistance (*kdr*), caused by mutations in the VGSC. Knockdown resistance was first identified in *Musca domestica*,^5^ with molecular analysis later identifying a leucine‐to‐phenylalanine substitution (L1014F) within the IIS6 transmembrane region conferring resistance.^6^ An enhanced form of *kdr*, known as *super‐kdr*, was also initially identified in *M*. *domestica* ^7^; molecular analysis implicated a methionine‐to‐threonine substitution in the IIS4–IIS5 loop as responsible for the more potent resistance phenotype.^6^ The L1014F substitution is widespread: a 2013 review reported this specific mutation in more than 20 pest species, and when other substitutions at L1014 are included the number exceeds 30 pest species.^8^

Among key cereal aphid pests, pyrethroid resistance has been reported worldwide in *Sitobion avenae*,^9^ *Metopolophium dirhodum* ^10^ and *Rhopalosiphum padi*.^11^ In Europe, however, *S*. *avenae* is the only cereal aphid species reported to carry *kdr*‐mediated resistance to pyrethroids; resistant populations have been detected in the UK,^9^, ^12^ Ireland^13^ and France.^14^ Reduced sensitivity to pyrethroids has been observed in *R*. *padi*, but these cases have been attributed to metabolic resistance rather than *kdr*, and to date there is no evidence of pyrethroid resistance in European populations of *M*. *dirhodum*. In *S*. *avenae*, *kdr* resulting from the L1014F mutation has been found at relatively low frequencies in field surveys^12^, ^13^, ^15^; a fitness cost identified in the dominant resistant clone compared with a susceptible clone may help explain this low frequency.^16^ Although pyrethroid‐resistant populations of *R*. *padi* carrying a *super‐kdr* mutation (M918L) have been reported in China,^11^ *super‐kdr* has not been reported in European cereal aphids, and no VGSC mutation other than L1014F had previously been reported for the English grain aphid, *S*. *avenae*.

In this study, we genetically screened key regions of the VGSC (IIS4–IIS5 loop and IIS6) known to harbour resistance‐conferring mutations. Specimens were sampled across multiple locations and years in Ireland, enabling us to detect individuals carrying the L1014F mutation and to screen for additional mutations, including those associated with *super‐kdr*.

## 2 MATERIALS AND METHODS

### 2.1 English grain aphid collection

The English grain aphid samples used in this study were collected from three 12.2‐m suction towers located in Ireland at Oak Park (Carlow), Ashtown (Dublin) and Crowbally, Castlemartyr (Cork). The suction‐traps continuously measured the aerial density of flying aphids, sampling at the logarithmic mean height of aphid flight (12.2 m). The suction towers collected insects daily, with samples preserved in ethanol (50%), brought into the laboratory on a weekly basis and refrigerated until processing. Samples were separated into aphids and other insects under a stereomicroscope, and the English grain aphid was identified to species level using the taxonomic keys of Blackman (2010).^17^ The specimens were placed in ethanol (99%) and stored at −70 °C until further testing. A subsample of grain aphids from the three towers across 2021–2024 were selected for testing here (Supporting Information, Table S1).

### 2.2 Extraction of RNA from aphid specimens and complementary DNA synthesis

Aphid specimens were removed from the −70 °C freezer and aphids were separated into individual wells of a Lysing Matrix Z 96‐well rack (MP Biomedicals, CA, USA). Each well was pre‐filled with 1.5–2 mm beads (yttria‐stabilised zirconium oxide). Prior to placing in the well, individual aphids were washed briefly in molecular grade H_2_O to remove ethanol and dried by placing on tissue paper. RNA was isolated from individual aphids using a Quick‐RNA 96 kit (Zymo Research, CA, USA) and complementary DNA (cDNA) was synthesised with Random Hexamer Primer and RevertAid reverse transcriptase. Full protocol details can be found at Ballandras et al.^18^

### 2.3 Amplicon sequencing including regions of the VGSC gene

The aphids were genotyped using a multiplex‐polymerase chain reaction (PCR) amplicon sequencing based on the GT‐seq approach originally described by Campbell et al.^19^ This assay was designed to facilitate the genotyping of *S. avenae*, identification of the L1014F mutation, the screening of regions harbouring additional mutations known to infer insecticide resistance in other species, and detection of YDV in aphids. In brief, it was originally designed to target 42 regions in the grain aphid genome^20^ and 19 regions in the genome of YDV^21^. The primers were designed with the aid of genome re‐sequencing data of the English grain aphid (Bio Project PRJNA1039626) and full protocol details can be found in Ballandras et al.^18^ Although targets for YDV were included, the resulting sequencing data were not analysed as part of this study; the full data are available in the National Center for Biotechnology Information (NCBI) as described below. Three amplicons were targeted in the VGSC gene, two covering the L1014F mutation and one covering the so‐called *super‐kdr* region. One of the targets covering the L1014F mutation was designed to cross an intron (successful amplification from cDNA, AM_01 in Fig. 1) and in the other case, a primer was placed in the intron (successful amplification from DNA).

**Figure 1:**
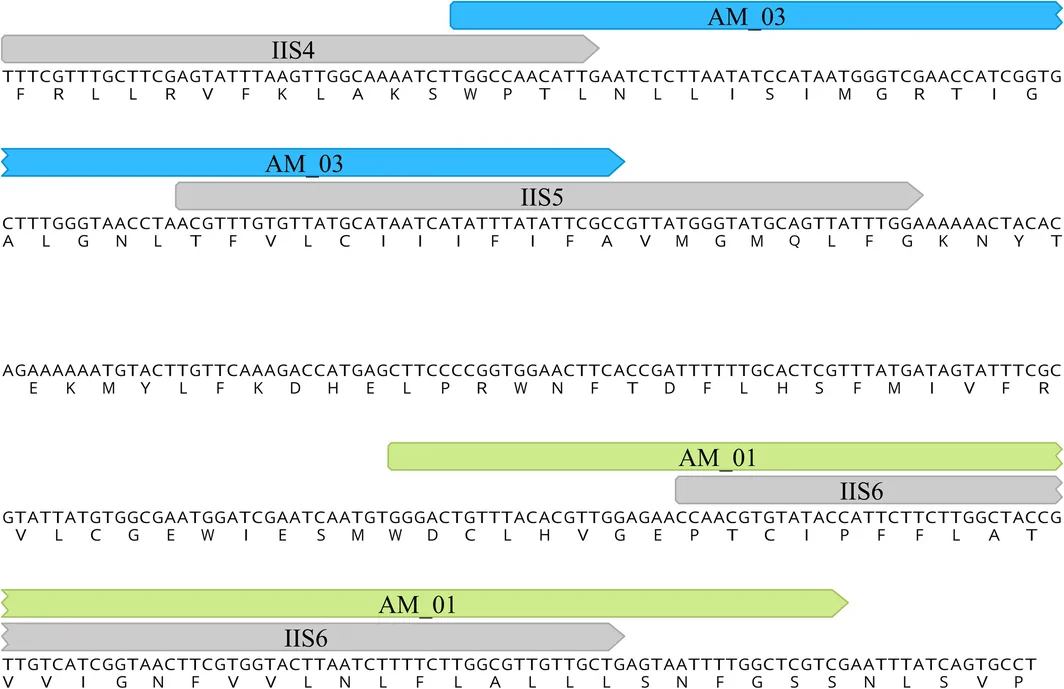
The regions in the VGSC gene amplified in the assay. AM_03 covers the region where additional mutations are associated with *kdr* (e.g. *super‐kdr* M918T/L) and AM_01 and AM_02 that cover the region with the L1014F mutation. The AM_02 amplicon is not shown but overlaps with AM_01 until after the L1014F mutation where the reverse primer is in the intron (see Supporting Information, Fig. S1 for full details). The AM_01 and AM_03 regions highlighted are amplicons after removing forward and reverse primers.

The third target was designed to amplify a segment that covers mutations previously identified as being associated with *super*‐*kdr* in other species (AM_03 in Fig. 1). The libraries were purified using AMPure XP magnetic beads (A63881; Beckman Coulter, Brea, CA, USA) and sequenced with Illumina paired‐end 150 bp reads (NovoGene, UK).

### 2.4 Analysis of sequencing data

Sequence data paired‐end reads were first merged using flash2,^22^ and merged reads were aligned to the *S*. *avenae* genome^20^ using bwa.^23^ The genome was supplemented with a specific cDNA gene fragment targeted by the AM_01 primer pair (this primer pair crosses an intron). Sequencing data were deposited in the NCBI short read archive and can be accessed through BioProject PRJNA1330828 and PRJNA1311848. Read‐backed haplotype analysis was carried out using SMAP (v. 5.0.6) *haplotype‐window*.^24^ The interval bounds selected for discrete calls were defined from haplotype frequencies: values of 0–0.1 were considered homozygote for the reference allele, 0.1–0.9 heterozygote, and 0.9–1 homozygote for the alternate allele. The minimum haplotype frequency was set to 25 (−f 25) which required that a haplotype was present at a frequency of at least 25% in at least one sample (expected in a diploid). The minimum read count at a locus was set to 30.

The completeness of loci was assessed, and data associated with the targets in the VGSC gene were extracted for further analysis. Data associated with remaining targets in the aphid genome were also extracted for further analysis of *S. avenae* diversity. SMAP *relatedness pairwise* ^25^ was run with the following settings: ‐sc 10 and ‐lc 0.6, creating a pairwise Jaccard genetic similarity matrix. Further data analysis and visualisation was carried out in R^26^ using the packages *tidyverse*,^27^ *vegan*,^28^ *ggraph* ^29^ and *igraph*.^30^ Similarity graphs were plotted based on the Jaccard similarity matrix with a threshold of 0.95 for forming edges between nodes, and using the force‐directed Fruchterman–Reingold layout algorithm.^31^ Data associated with targets in YDV were not analysed further as part of this study.

### 2.5 Validation of newly identified mutations

The newly identified mutations in the region of the VGSC gene covering the *super‐kdr* region were validated by going back to the extracted RNA from the individual specimens with these mutations and carrying out reverse transcription polymerase chain reaction (RT‐PCR) using the primers targeting this region (AM_03 forward: 5′‐ CTTCGAGTATTTAAGTTGGCAAAATC‐3′ and reverse: 5′‐TTTCCAAATAACTGCATACCCATAAC‐3′). The reactions were set up using PrimeTime One‐Step Master Mix (Integrated DNA Technologies, IA, USA) and the recommended standard cycling protocol. The PCR products were sent for premium PCR sequencing, which was performed by Plasmidsaurus using Oxford Nanopore Technology (ONT).

## 3 RESULTS

### 3.1 English grain aphid diversity

In addition to the targets amplifying regions of the VGSC gene, 32 other regions throughout the grain aphid genome were successfully amplified (read data generated) as part of the multiplex PCR assay. In many of these cases, locus completeness was >0.75 (Supporting Information, Fig. S2), as expected for high read‐depth sequencing of an amplicon library. Loci with completeness <0.75 were removed (5 of the 32 successfully amplified targets), and the number of haplotypes identified in the remaining loci ranged from 12 to 27 (Supporting Information, Fig. S3). At most loci, one to two haplotypes dominated (Supporting Information, Fig. S4). There were clusters of specimens observed based on the haplotype data (Fig. 2) with the largest cluster based on a Jaccard similarity >0.95 having 110 specimens.

**Figure 2:**
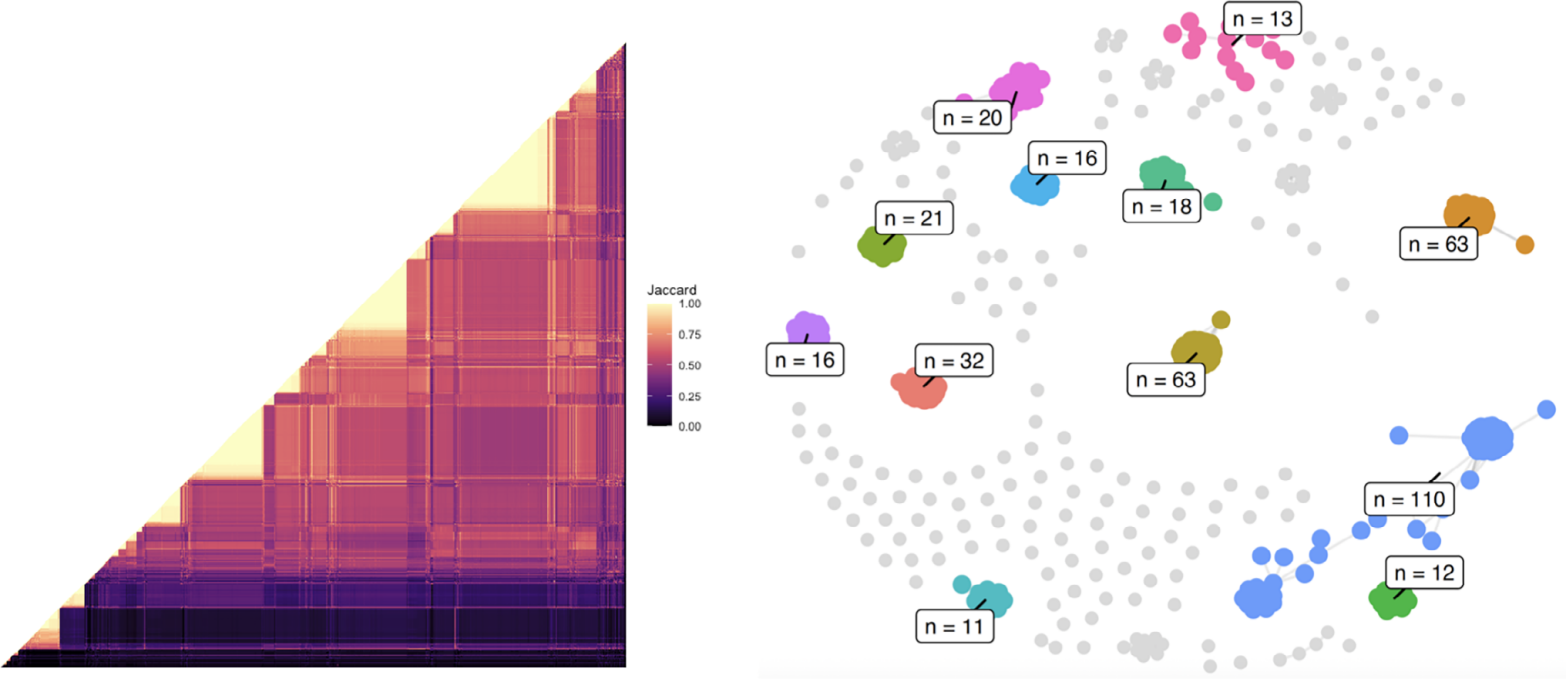
Jaccard similarity of grain aphid specimens genotyped in this study. The Jaccard similarity matrix based on haplotypes from the 27 successfully amplified targets is shown on the left. Shown on the right are samples represented as a similarity network where nodes correspond to individual specimens and edges connect pairs with a Jaccard similarity threshold >0.95. The clusters with more than ten specimens are coloured and specimen numbers in these clusters are labelled.

### 3.2 Molecular screening of VGSC gene for L1014F mutation

Screening for the L1014F mutation was conducted using the amplicon targets AM_01 and AM_02. AM_01 is designed to cross an intron and will only amplify the required product with cDNA as the template, whereas AM_02 will amplify the required product from DNA. The L1014F region was successfully amplified with both targets in 143 samples, with AM_01‐only in 54 samples, AM_02‐only in 169 samples and failed at both targets for 260 samples. This resulted in the ability to determine the presence/absence of the L1014F mutation in 366 samples. In cases in which the region was genotyped with both targets, there was good agreement with an accuracy of 0.98 with only three false positives identified. In all three cases, we favoured the results of the AM_02 target because there is greater support owing to a higher read depth.

AM_01 resulted in the identification of seven amplicon sequence variant (ASV) haplotypes, two of which harboured the nucleotide substitution leading to the L1014F mutation (Fig. 3; AM01.H03 and AM01.H07). Given that most nucleotide substitutions were synonymous, these seven ASV haplotypes only translated to two amino acid haplotypes, one of which has the L1014F mutation (Fig. 3).

**Figure 3:**
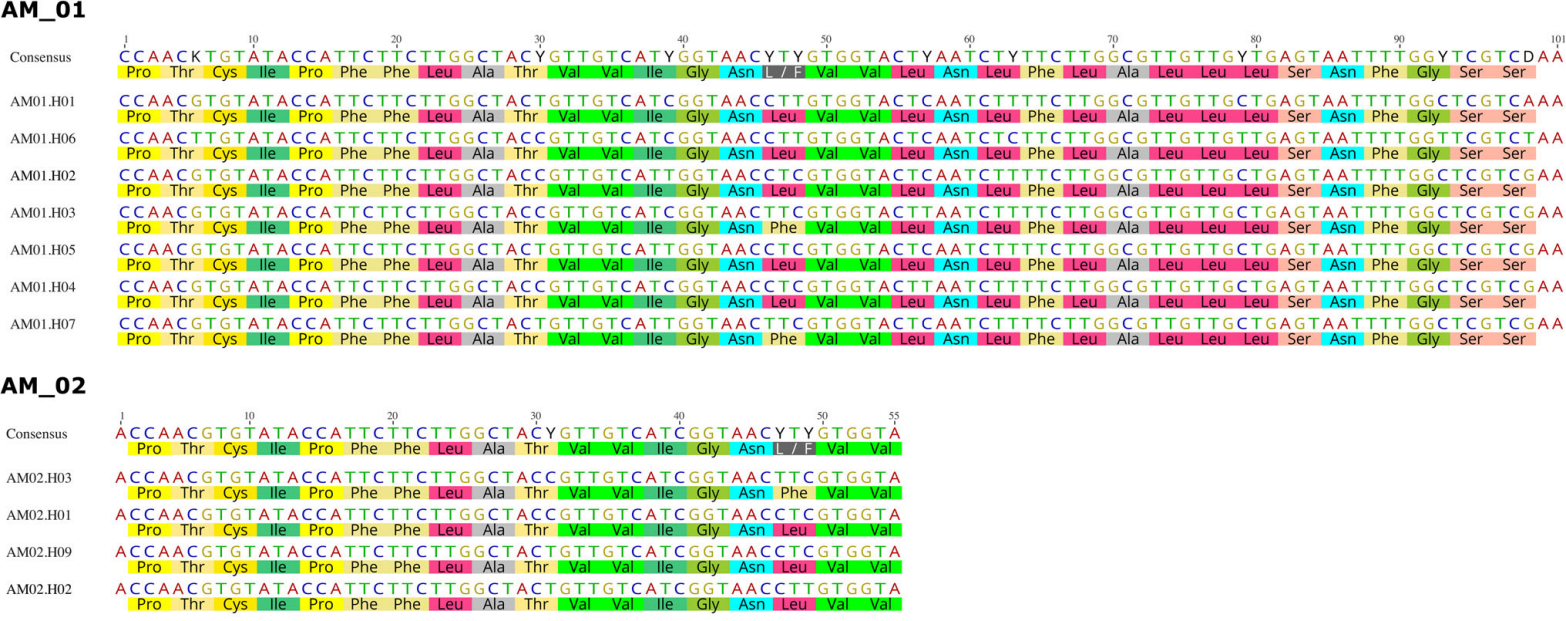
AM_01 and AM_02 haplotypes and amino acid sequences. The consensus amino acid sequence is shown on top. The AM_02 haplotype sequence has been truncated before the intron and non‐redundant haplotype sequences after truncation were removed.

AM_02 resulted in the identification of four ASV haplotypes, one of which harboured the nucleotide substitution leading to the L1014F mutation (Fig. 3). Given that most nucleotide substitutions were synonymous, these four ASV haplotypes translated to only two amino acid haplotypes, one of which has the L1014F mutation (Fig. 3).

Across all specimens in which L1014F status could be successfully determined, we identified 65 carrying this mutation in suction towers between 2022 and 2024, with L1014F‐resistant *S*. *avenae* identified across all regions and years (Fig. 4). The majority of these were in a single cluster (Jaccard similarity >0.95) (Fig. 4). The cluster with the majority of L1014F‐resistant *S*. *avenae* also has a specimen from a laboratory colony of the clone SA3 (Supporting Information, Fig. S5).

**Figure 4:**
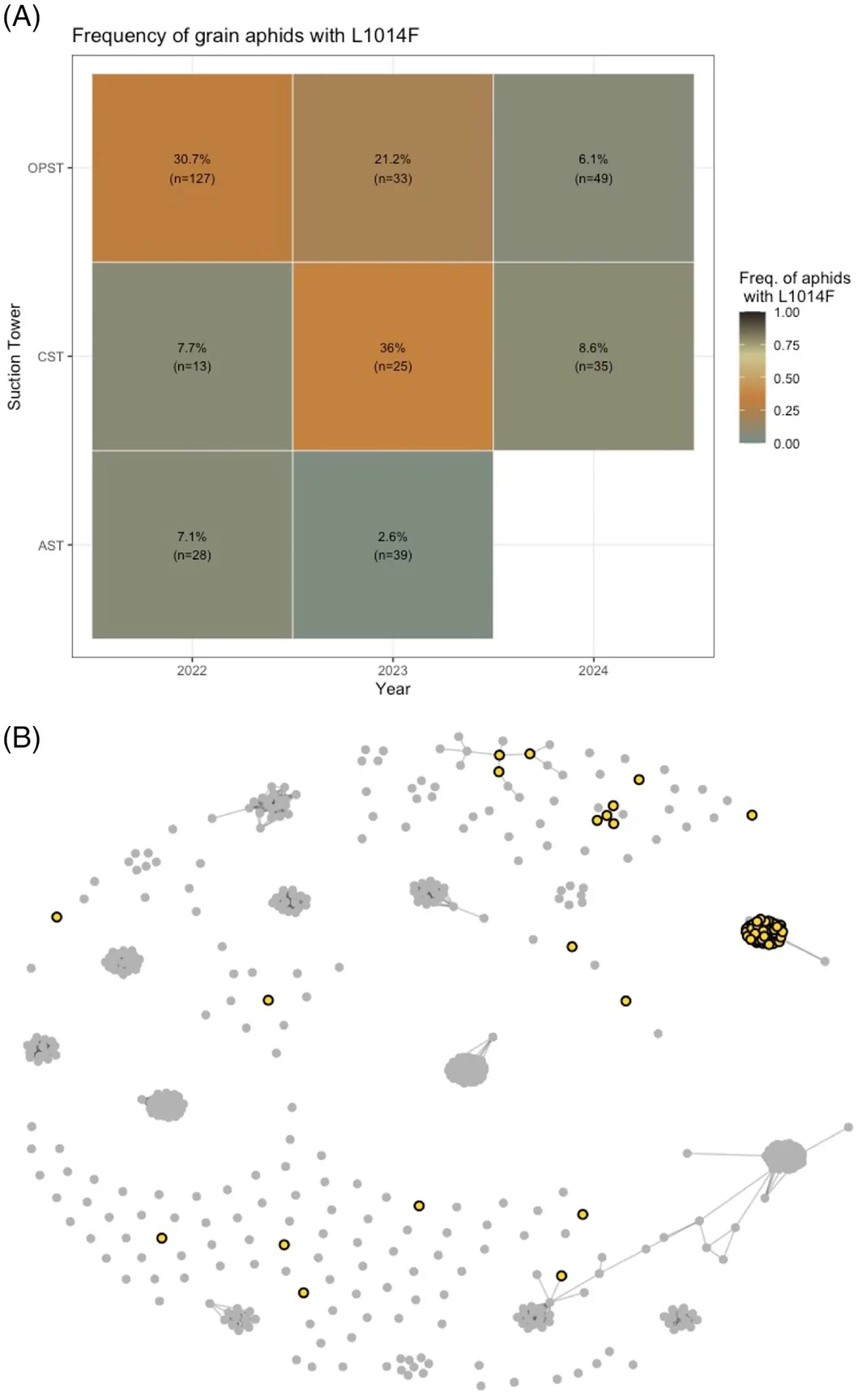
Summary of L1014F mutation screening. (A) The proportion of English grain aphids tested that possessed an allele carrying the L1014F mutation from 2022 to 2024 and across the three suction towers where specimens were sourced. (B) Samples are represented as a similarity network where nodes correspond to individual specimens and edges connect pairs with a Jaccard similarity threshold >0.95. Specimens with L1014F have been highlighted in yellow.

### 3.3 Molecular screening of the *super‐kdr* region of the VGSC gene

The region with *super‐kdr* was successfully amplified and sequenced for 539 specimens, resulting in the identification of 24 ASV haplotypes. One of these haplotypes was dominant, being homozygous in 451 specimens and heterozygous in 27 for an allele frequency of 0.862 (Supporting Information, Table S2).

Two ASV haplotypes had the M918L mutation, identified in three specimens (Fig. 5) two from 2022 and one from 2023. In all three specimens, the M918L mutation existed in a heterozygous state. In the case of AM03.H16, the M918L mutation was caused by an A to C mutation in the first position of the codon and in the case of AM03.H23, the M918L mutation was caused by an A to T mutation in the first position of the codon.

**Figure 5:**
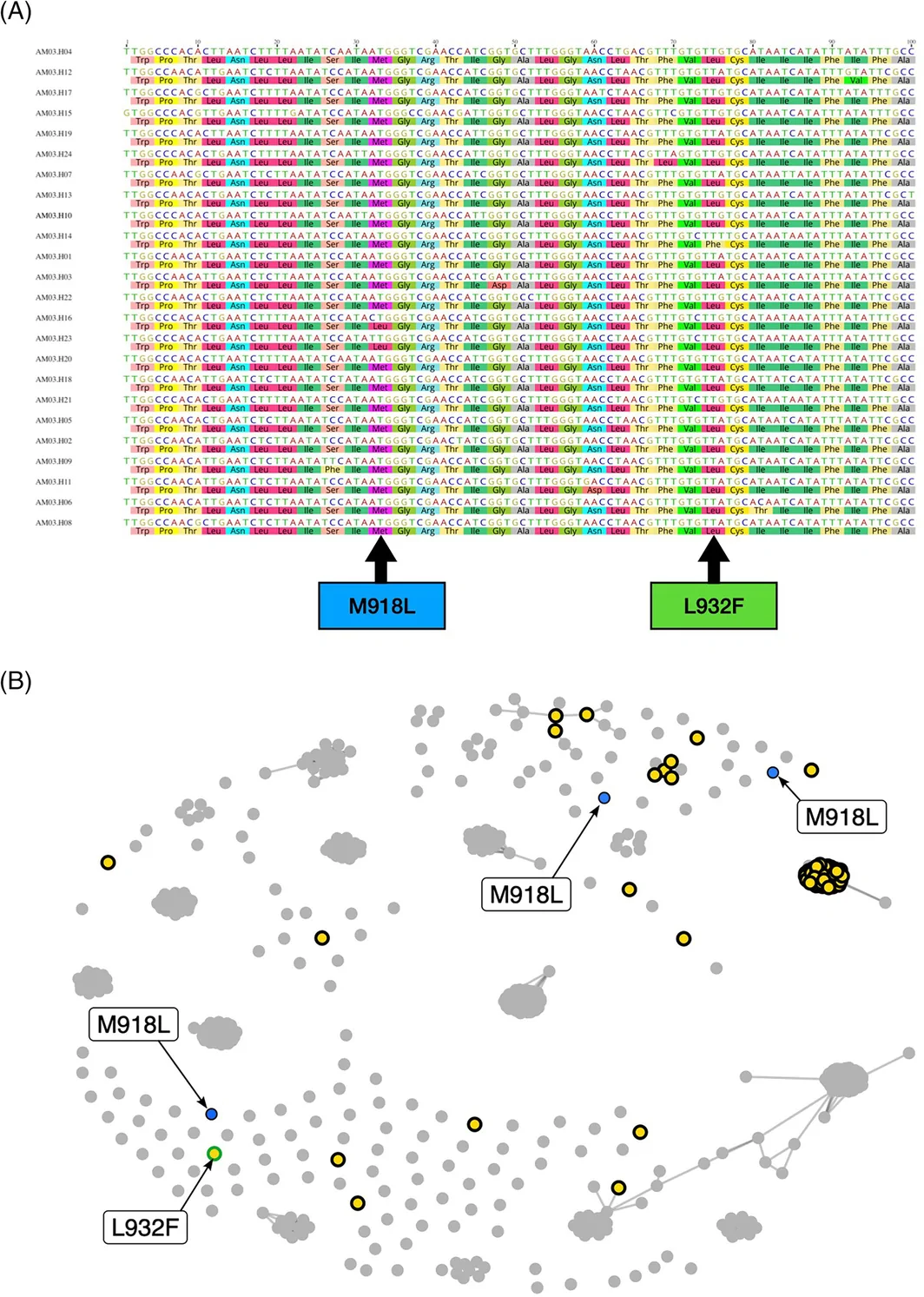
(A) *Super‐kdr* haplotypes identified across 539 specimens successfully genotyped at this locus. (B) The location of the M918L and L832F mutations are highlighted in alignment and location in clusters (samples are represented as a similarity network where nodes correspond to individual specimens and edges connect pairs with a Jaccard similarity threshold >0.95). Specimens with the L1014F mutation are denoted yellow, the M918L mutation s shown in blue, and the single specimen with both the L932F and L1014F mutations is indicated by yellow with a green outer circle.

In addition to M918L, another haplotype with the mutation L932F was identified in a single specimen. The specimen was heterozygous at this locus and identified in a sample from 2022. This specimen also carried the L1014F mutation (Fig. 5).

The M918L and L932F mutations were both confirmed in the four specimens using the originally extracted RNA and the AM_03 primers in a one‐step RT–PCR, followed by ONT sequencing at high depth (Supporting Information, Fig. S6 and Table S3). Additional non‐synonymous mutations were identified at this locus in individual specimens that had not been identified previously in other insect species (Fig. 5); however, these were not validated with high‐depth ONT sequencing.

## 4 DISCUSSION

Maintaining a selection of viable control options is fundamental to both pesticide resistance management and integrated pest management. The current lack of suitable alternative pesticides for cereal aphid control increases the risk of selection for pyrethroid resistance. This risk is already evident in the English grain aphid, *S. avenae*, where *kdr* was first reported in the UK,^9^ and has since been recorded in Ireland^32^ and France.^14^ The L1014F substitution conferring *kdr*, even when found in the heterozygous state, has been associated with an almost 40‐fold increased resistance to the type II pyrethroid lambda‐cyhalothrin.^9^ In this study we report the occurrence of the *super*‐*kdr* mutation M918L in the VGSC of *S*. *avenae* sampled from migrating aphids captured in 12.2‐m suction towers in Ireland during 2022 and 2023. We also detected the L932F substitution co‐occurring with L1014F from a specimen collected in 2022. These findings may have implications for the future efficacy of pyrethroids and should inform resistance management strategies for cereal crops.

The frequency of the L1014F substitution in samples from Irish suction towers between 2022 and 2024 is consistent with frequencies reported from other studies in Ireland,^15^, ^33^ and the UK,^12^ and again we found no strong evidence of an increase in *kdr* following the ban on neonicotinoid seed treatments for outdoor use and the consequent greater reliance on pyrethroids. This persistence of *kdr* at current levels may reflect the documented fitness costs associated with L1014F, including longer development time, reduced lifespan, lower asexual reproductive output and greater susceptibility to parasitoids.^16^, ^34^ Furthermore, the importance of untreated refugia for susceptible clones together with fitness costs may help limit the dominance of resistance; this may be of particular importance for the English grain aphid in landscapes dominated by grass lands such as Ireland, where pastures act as a refuge from crops under spray.

Most L1014F‐carrying specimens clustered with a laboratory‐maintained SA3 clone; this clone, defined by SSR markers, is considered the dominant *kdr* lineage in the UK and Ireland. Nevertheless, L1014F was also detected in specimens that did not cluster with SA3, echoing earlier microsatellite‐based findings.^13^ *S*. *avenae* with L1014F were found in all sampled years and regions. The phenotypic consequences of *kdr* homozygosity remain unknown because homozygous *kdr* grain aphids have not yet been detected in the field, preventing direct pyrethroid sensitivity assays; importantly, however, the L1014F mutation does not preclude production of sexual morphs,^32^ so sexual crosses among *kdr* heterozygotes could potentially generate homozygous offspring.

Our analysis identified two VGSC substitutions previously implicated in reducing pyrethroid sensitivity in other species, but not previously reported in *S*. *avenae*. One of these represents an enhanced form of *kdr*, known as *super‐kdr*, which was first identified in *M*. *domestica* ^7^ and classically represented by a methionine‐to‐threonine substitution in the IIS4–IIS5 loop (M918T). This mutation was later identified in *M*. *persicae* ^4^ and, when it co‐occurs with L1014F, typically produces very high resistance to both type I and type II pyrethroids.^35^ A different amino acid substitution at the same site, a methionine‐to‐leucine substitution (M918L), has since been detected in *M*. *persicae*,^36^ *Aphis gossypii* ^37^ and *R*. *padi*.^11^ We identified the methionine‐to‐leucine substitution in the heterozygous state at this position in three aphid specimens: two from the Carlow suction tower (2022) and one from the Dublin suction tower (2023). The two specimens from Carlow carried the same allelic makeup at this locus, whereas the specimen from Dublin had a different allelic makeup. In both cases the mutations were identified in samples collected in June. Studies in *R*. *padi* indicate that the M918L substitution confers high resistance to type I and type II insecticides, in particular extremely high resistance to the type II lambda‐cyhalothrin (194.7‐fold).^38^ However, it is also associated with reduced longevity and fecundity in the absence of insecticide selection, indicating a fitness cost.^38^ Unlike the M918T variant, M918L can occur more commonly as a single polymorphism without L1014F,^36^ and in all three cases here the substitution did not co‐occur with L1014F. The amino acid substitution M918L has been previously identified in the aphids *A. gossypii* ^39^ and *R*. *padi*,^11^, ^40^ where it is associated with acute resistance to lambda‐cyhalothrin, a key component of pyrethroid insecticides. In both *R*. *padi* and *A*. *gossypi*, the substitution results from a single nucleotide change at codon 918, changing ATG to TTG; this specific mutation was identified in the specimen from Dublin (2023). By contrast, the two specimens from Carlow (2022) carried the ATG to CTG change, which also encodes the same amino acid substitution. This mutation has not previously been observed in *S*. *avenae*, and the acute resistance it may confer requires empirical validation. Nevertheless, the presence of M918L in other aphid species, where it has been associated with pyrethroid resistance under similar selection pressures, suggests that it may likewise confer resistance to lambda‐cyhalothrin in the English grain aphid.

We also identified a leucine‐to‐phenylalanine substitution at the position corresponding to the previously identified L932F substitution in *M*. *persicae* ^36^ and head lice.^41^ In *M*. *persicae*, the mutation was found in combination with L1014F; the single specimen in our study that carried this substitution also carried L1014F. This specimen was collected from a Carlow suction trap in June 2022. In *M*. *persicae* it was not possible to characterise the resistance phenotype associated with the L1014F + L932F combination, and because we do not have live *S*. *avenae* carrying L932F, we likewise cannot estimate its potential impact on pyrethroid sensitivity in the grain aphid. Wide field sampling around the Carlow and Dublin suction tower catchments is therefore required to determine the prevalence and establishment of clones carrying L932F or M918L. Subsequent insecticide sensitivity bioassays should then assess any reduction in pyrethroid susceptibility, and fitness‐cost experiments (as performed for L1014F clones) are needed to predict whether such clones are likely to predominate or be constrained by reduced fitness. Notably, L1014F was detected retrospectively in UK suction tower samples before it manifested as operational spray failures, so continued vigilance for these and other emergent mutations is warranted.

In summary, we have identified a *super‐kdr* variant, M918L, along with an L932F variant in the English grain aphid. Because these were identified in archived samples, their impact on resistance in the grain aphid is unknown; however, based on observations in other aphid species (including cereal aphids), they are likely to affect resistance phenotypes. Regular monitoring of the incidence and spread of known and emerging insecticide‐resistant clones is important both to preserve the effectiveness of our limited insecticide arsenal and to determine when their field efficacy may no longer be sufficient for control. Suction towers provide valuable, management‐independent surveillance that can enable early detection of resistance mechanisms and problematic clones, thereby guiding more intensive field assessments where necessary.

## CONFLICT OF INTEREST

The authors declare that they have no competing financial interests.

## Supporting information


**Figure S1.** Full details of the primers used to amplify the three regions targeted in the VGSC gene (AM_01, AM_02, and AM_03).
**Fig. S2**. Locus completeness plot ‐ the percentage of samples that have data (after applying filters) on a per locus level. Given this is high depth sequencing of a small amplicon set, the majority of samples should have many high completeness loci and few low completeness loci.
**Fig. S3**. Haplotype diversity at each locus – the number of haplotypes observed in the population at each locus (loci with completeness below 0.75 were removed – see Fig. S1).
**Fig. S4**. Haplotype frequency at each locus – the frequency of each haplotype at each locus is shown. It can be seen that all loci have one or two haplotypes that are dominant in the specimens studied here.
**Fig. S5**. Clusters based on Jaccard similarity greater than 0.95. Aphids with L1014F are shown in yellow and two control lab colonies (SA27 without L1014F and SA3 with L1014F) are highlighted.
**Fig. S6**. Validation of mutations M918L and L932F identified through the initial pooled multiplex amplicon sequencing. The validation started from extracted RNA from individual specimens and carried out RT‐PCR followed by ONT sequencing. The results are represented as graph showing proportion of reads supporting a base; in excess of 8 K reads at each locus and absolute allele counts are shown in Supplementary Table S3.
**Table S1**. The source of aphids subsampled for testing in this study. AST is the suction tower in Ashtown (Dublin), OPST is the suction tower in Oak Park (Carlow), and CST is the suction tower in Crowbally, Castlemartyr (Cork).
**Table S2**. The haplotype allele frequency at AM_03 amplifying the *super‐kdr* region of the VGSC gene.
**Table S3**. Validation of the mutations in the *super‐kdr* region of the VGSC gene by amplification and high depth ONT sequencing in individual specimens.

## Data Availability

The data that support the findings of this study are openly available in NCBI SRA at https://www.ncbi.nlm.nih.gov/bioproject/, reference number PRJNA1330828, PRJNA1311848.
