# Supplementary material for: The first report of M918L ( super‐kdr ) and L932F voltage‐gated sodium channel mutations in the English grain aphid ( Sitobion avenae )
Source: Pest Manag Sci. 2026 Aug 5;82(12):11844–52. doi: 10.1002/ps.71178 (PMC13756813; doi:10.1002/ps.71178)
Supplement: Supplementary file 1 [file PS-82-11844-s001.docx]

**Title:** The first report of M918L (*super-kdr*) and L932F Voltage-Gated Sodium Channel mutations in the English grain aphid (*Sitobion avenae*).

**Short running title:** VGSC mutations (M918L and L932F) in the English grain aphid

Virgile Ballandras^1,2^, Louise McNamara^1^, James Carolan^2^, Tom Ruttink^3,4^, Stephen Byrne^1^

^1^Teagasc, Crops Research Centre, Oak Park, Carlow R93XE12, Ireland

^2^Department of Biology, Maynooth University, Maynooth, Co. Kildare W23 F2H6, Ireland

^3^Flanders Research Institute for Agriculture, Fisheries and Food (ILVO), Plant Sciences Unit, Caritasstraat 39, 9090, Melle, Belgium

^4^Department of Plant Biotechnology and Bioinformatics, Faculty of Sciences, Ghent University, Technologiepark 71, 9052 Ghent, Belgium

**Keywords:** knockdown resistance; English grain aphid; M918L; L932F; super-kdr

**Supplemental Figure S1:** Full details of the primers used to amplify the three regions targeted in the VGSC gene (AM_01, AM_02, and AM_03).

**Supplemental Figure S2:** Locus completeness plot - the percentage of samples that have data (after applying filters) on a per locus level. Given this is high depth sequencing of a small amplicon set, the majority of samples should have many high completeness loci and few low completeness loci.

**Supplemental Figure S3:** Haplotype diversity at each locus – the number of haplotypes observed in the population at each locus (loci with completeness below 0.75 were removed – see supplemental figure S1).

**Supplemental Figure S4:** Haplotype frequency at each locus – the frequency of each haplotype at each locus is shown. It can be seen that all loci have one or two haplotypes that are dominant in the specimens studied here.

**Supplemental Figure S5:** Clusters based on Jaccard similarity greater than 0.95. Aphids with L1014F are shown in yellow and two control lab colonies (SA27 without L1014F and SA3 with L1014F) are highlighted.

**Supplemental Figure S6:** Validation of mutations M918L and L932F identified through the initial pooled multiplex amplicon sequencing. The validation started from extracted RNA from individual specimens and carried out RT-PCR followed by ONT sequencing. The results are represented as graph showing proportion of reads supporting a base; in excess of 8K reads at each locus and absolute allele counts are shown in Supplementary Table S3.

**Supplementary Table S1:** The source of aphids subsampled for testing in this study. AST is the suction tower in Ashtown (Dublin), OPST is the suction tower in Oak Park (Carlow), and CST is the suction tower in Crowbally, Castlemartyr (Cork).

|  |  | **Month** | | | | | | | | |
| --- | --- | --- | --- | --- | --- | --- | --- | --- | --- | --- |
| **Year** | **Suction Tower** | ***Jan*** | ***Apr*** | ***May*** | ***Jun*** | ***Jul*** | ***Aug*** | ***Sept*** | ***Oct*** | ***Nov*** |
| 2021 | AST | 0 | 0 | 0 | 0 | 0 | 0 | 0 | 1 | 0 |
| 2021 | OPST | 0 | 0 | 1 | 0 | 0 | 0 | 0 | 0 | 0 |
| 2022 | AST | 0 | 0 | 2 | 2 | 3 | 18 | 3 | 3 | 1 |
| 2022 | CST | 0 | 0 | 3 | 0 | 12 | 10 | 1 | 0 | 0 |
| 2022 | OPST | 0 | 0 | 5 | 38 | 98 | 1 | 1 | 1 | 0 |
| 2023 | AST | 0 | 1 | 11 | 45 | 0 | 0 | 0 | 0 | 0 |
| 2023 | CST | 0 | 0 | 4 | 5 | 11 | 12 | 2 | 1 | 0 |
| 2023 | OPST | 0 | 0 | 4 | 53 | 12 | 1 | 1 | 0 | 0 |
| 2024 | CST | 0 | 1 | 1 | 21 | 86 | 1 | 2 | 4 | 1 |
| 2024 | OPST | 1 | 2 | 6 | 15 | 68 | 27 | 4 | 1 | 0 |

**Supplementary Table S2:** The haplotype allele frequency at AM_03 amplifying the *super-kdr* region of the VGSC gene.

| **HapName** | **homozygous** | **heterozygous** | **allele_copies** | **frequency** |
| --- | --- | --- | --- | --- |
| AM03.H01 | 451 | 27 | 929 | 0.862 |
| AM03.H02 | 33 | 7 | 73 | 0.068 |
| AM03.H10 | 3 | 13 | 19 | 0.018 |
| AM03.H19 | 5 | 0 | 10 | 0.009 |
| AM03.H17 | 4 | 0 | 8 | 0.007 |
| AM03.H13 | 3 | 0 | 6 | 0.006 |
| AM03.H04 | 2 | 0 | 4 | 0.004 |
| AM03.H21 | 0 | 4 | 4 | 0.004 |
| AM03.H08 | 1 | 1 | 3 | 0.003 |
| AM03.H05 | 1 | 0 | 2 | 0.002 |
| AM03.H07 | 1 | 0 | 2 | 0.002 |
| AM03.H15 | 1 | 0 | 2 | 0.002 |
| AM03.H16 | 0 | 2 | 2 | 0.002 |
| AM03.H18 | 1 | 0 | 2 | 0.002 |
| AM03.H20 | 1 | 0 | 2 | 0.002 |
| AM03.H24 | 1 | 0 | 2 | 0.002 |
| AM03.H03 | 0 | 1 | 1 | 0.001 |
| AM03.H06 | 0 | 1 | 1 | 0.001 |
| AM03.H09 | 0 | 1 | 1 | 0.001 |
| AM03.H11 | 0 | 1 | 1 | 0.001 |
| AM03.H12 | 0 | 1 | 1 | 0.001 |
| AM03.H14 | 0 | 1 | 1 | 0.001 |
| AM03.H22 | 0 | 1 | 1 | 0.001 |
| AM03.H23 | 0 | 1 | 1 | 0.001 |

**Supplementary Table S3:** Validation of the mutations in the *super-kdr* region of the VGSC gene by amplification and high depth ONT sequencing in individual specimens.

| Specimen | Mutation | Allele 1 | Allele 1 read counts | Allele 2 | Allele 2 read counts |
| --- | --- | --- | --- | --- | --- |
| P2022_006_E4 | M918L | CTG | 4,121 | ATG | 3,908 |
| P2022_006_G3 | M918L | CTG | 3,979 | ATG | 4,042 |
| P2023_011_E9 | M918L | TTG | 4,262 | ATG | 3,757 |
| P2022_006_F2 | L932F | TTT | 4,094 | TTG | 3,933 |
